# From depression to wellbeing: How to protect the mental health of isolated people under the “dynamic clearance” of patients with COVID-19

**DOI:** 10.3389/fpsyg.2023.1124063

**Published:** 2023-02-16

**Authors:** Yuntao Bai, Shuai Ma

**Affiliations:** School of Business, Shandong Management University, Jinan, China

**Keywords:** mental health, differential game, psychological counseling by the government, psychological counseling of social forces, self-counseling

## Abstract

In 2020, COVID-19 became a global pandemic. The Chinese government’s quarantine measures tend to cause anxiety, tension and depression among the quarantined people. This article constructs a differential game model of self-regulation, government guidance and social forces guidance. Then, the psychological benefits of the masses and the benefits of the whole society under the three modes are obtained, and the applicable conditions of various connection modes are compared. The research results show that: compared with social power channeling, the public under the government channeling mode can obtain more psychological benefits. However, with the increase of guidance, the difference between the psychological benefits of different ways of guidance first decreases and then tends to be stable. Under the guidance mode, the social benefits of the government decrease, and the more guidance, the smaller the social benefits. Therefore, both the government and social forces should make use of limited resources to conduct appropriate psychological counseling for the isolated population.

## 1. Introduction

### 1.1. Background and research significance

The number of people infected with COVID-19 has increased dramatically since early 2020. On 11 March 2020, WHO declared the novel coronavirus pneumonia a global pandemic. The COVID-19 pandemic has had a significant impact on the mental health of the public. According to the epidemic mental health report released by One Psychology, 51.52% of the country is in a good state of mind. In Hubei Province, the worst-hit province in 2020, only 42.25% reported good mental health. However, in Liaoning Province, which was less affected by the epidemic, 58.78% were in good mental condition. Under the “dynamic clearance” of patients with COVID-19, the Chinese government often has to take isolation measures. How to solve the psychological problems of these isolated people is related to the psychological health of the public society.

Although China has managed to contain the coronavirus as a whole, local outbreaks have occurred from time to time in the country. To effectively control the spread of COVID-19, China strictly implements the general policy of “dynamic zero clearance” (Burki, 2022). In the event of an outbreak in an area, the Chinese government will reduce the movement of people there, restrict residents in their communities, or place them in centralized quarantine. The isolation is divided into centralized medical quarantine and home quarantine. Centralized medical isolation means that suspected cases are completely isolated from the outside world (Hussain et al., 2022). After disinfection, only professional healthcare workers can enter the quarantine site. Infection control at quarantine sites and personal protection for medical professionals should be done (Aronna et al., 2021). Those quarantined at home should live independently, minimize contact with co-residents, clean and disinfect medical observation sites, and avoid cross-infection (Ingle et al., 2021).

The stability of a community has a very important impact on the mental health of residents (Ross et al., 2000). This way, though, can cut the chain of transmission and successfully control the spread of the epidemic. However, this approach is prone to social distancing, which may cause psychological problems for isolated persons (Peterson et al., 2021). Many quarantined people suffer from psychological problems such as tension, anxiety, and depression. To this end, various measures can be taken to alleviate the psychological problems of the isolated masses. Three common means include self-regulation, government psychological counseling, and social force psychological counseling. This can relieve the residents of anxiety, tension, depression, and other negative psychological adjustment issues. However, which method has more prominent psychological effects on residents is an important issue discussed in this article. In other words, each mode has its own advantages and disadvantages and scope of application. How to choose the right mode to realize the fastest speed of the isolated population from depression to ease of mind is the focus of this article.

### 1.2. Literature review

As a global pandemic, COVID-19 has a psychological impact on people. Many scholars have studied the psychological impact of COVID-19. For example, Khan and Khan (2021) analyzed the psychological impact of COVID-19 on people recovering from COVID-19, while Rosenbaum and Mincer (2021) analyzed the psychological needs of people recovering from COVID-19. Li et al. (2020) studied the impact of COVID-19 on students’ mental health. Lim et al. (2021) analyzed the impact of the COVID-19 pandemic on depression and anxiety in rheumatic patients. The abovementioned literature mainly studies the psychological impact of COVID-19 on different groups such as rehabilitated people, students, and rheumatic patients.

Quarantine measures can cause psychological problems even if some people have no contact with the virus. Some scholars have studied the psychological effects of isolation. For example, Lima et al. (2020) believe that long-term isolation can cause psychological damage. Alle and Berntsen (2021) suggested that isolation can lead to an increased risk of psychosis in residents. Isherwood et al. (2021) analyzed the psychological impact of isolation on residents during the COVID-19 pandemic through a telephone survey. Gierveld et al. (2018) analyzed that loneliness caused by isolation can have adverse effects on people’s physical resistance. The studies covered the major impacts of COVID-19 quarantines.

Both COVID-19 and quarantine measures can cause psychological problems. These psychological problems should be properly dealt with (Eisenbeck et al., 2021). To mitigate the psychological effects of isolation, some scholars have studied specific measures. For example, Hansen et al. (2021) believe that dance can reduce the psychological impact of the elderly during isolation. Henkel et al. (2020) believe that social robots can alleviate the loneliness of residents. Harris and Sandal (2020) argued that the role of trust in the healthcare system must be fully exploited.

The research includes the impact of COVID-19 on mental health, the impact of isolation on mental health, and how to deal with the psychological problems brought on by COVID-19. However, the daily number of COVID-19 infections and the areas under lockdown is dynamic, meaning that there are both new infections and recovered patients and new containment areas and noncontainment areas every day. The above studies do not reflect this dynamic process.

To make up for the shortcomings of the earlier studies, this article uses a differential game to study dynamic problems. At present, the differential game is mainly applied in the fields of pricing strategy (Chintagunta and Rao, 1996), environmental protection (Bai et al., 2022), advertising decision-making (Viscolani and Zaccour, 2009; Liu et al., 2012), and information security (Gao et al., 2013; Bandyopadhyay et al., 2014). Yin and Zhang (2021) used a differential game to study the joint prevention and control mechanism of COVID-19. Zhu et al. (2021) used a differential game to study the dynamic adjustment mechanism of mask emergency supply chain cooperation based on COVID-19. The earlier studies using differential game analysis to carry out the novel coronavirus epidemic did not analyze the ways the local government took to conduct psychological counseling for the quarantined masses.

The model and hypothesis in this article are based on the background of the novel coronavirus pandemic, and the Chinese government implements the policy of “dynamic clearance.” The policy can lead to psychological problems among the quarantined people. Effective relief of the negative emotions of the isolated population needs timely counseling. This article studies the problem of dynamic psychological counseling, i.e., what effect the government and social forces will have on the psychological counseling of isolated people. This article constructs a differential game model of self-guidance, government psychological guidance, and social force guidance. The optimal social and psychological benefits under various modes are obtained. The scope of application of various psychological counseling methods is compared and analyzed to provide a reference for effective psychological counseling to alleviate the psychological problems of the isolated population.

### 1.3. Problem description

Under the policy of dynamic zero clearance, the Chinese government quarantines close contacts and other people in order to effectively control the spread of the epidemic. People in isolation can become depressed, anxious, and nervous. It is an important problem how to relieve the bad psychological condition of isolated people and make them comfortable from depression. Therefore, the government and social forces will take certain measures to psychological isolation of the population. However, other research has focused on the dangers of COVID-19, the impact of isolation, and how to mitigate its impact. From the perspective of a dynamic game, this article analyzes the effect of different institutions in alleviating the negative psychology of the isolated population. This article summarizes the scope of the application of various psychological counseling methods.

For the sake of convenience, the game is divided into government, social forces, and residents. To effectively relieve the tension, anxiety, depression, and other adverse psychological states of local residents, the following three modes of psychological counseling and communication are mainly used:

1. Self-regulating mode. Once the epidemic comes, it will put a pause button on the local society. Since then, cities and roads have been closed, economic activity has been shaken, and countless people have been quarantined. This tends to cause psychological problems such as tension, anxiety, and depression among local residents. Sometimes, the isolation is too short for social forces and the government to evacuate residents. How to self-regulate is an important issue for isolated residents to face.
2. Government psychological counseling model. The COVID-19 outbreak is likely to cause psychological problems among residents. During the COVID-19 pandemic, the government has coordinated mental health forces and provided psychological services such as counseling and intervention for local residents to safeguard the mental health of the public. For example, the government’s Health Commission has set up assistance teams, including clinical psychiatric medical staff from local hospitals and volunteers qualified as psychological counselors, with certain work experience from psychological associations to form psychological rescue medical teams and psychological assistance hotline volunteer teams in order to provide psychological assistance, crisis intervention, and mental health publicity and education for the public. In addition, the National Health Commission of China issued the Guiding Principles for Emergency Psychological Crisis Intervention in the Outbreak of the Novel Coronavirus Pneumonia, which provides basic guidelines for the establishment of an effective social psychological counseling mechanism.
3. Psychological counseling model of social forces. As an important social force of psychological counseling, the psychological association can play an important role in alleviating the bad mood of local residents. To reduce depression, stress, and anxiety, local residents can communicate with psychological associations. Psychological associations and other social forces can give full play to the role of professional social work teams in the field of mental health and provide targeted services such as assistance, psychological counseling, spiritual comfort, and relationship adjustment for community residents. For example, in the early days of the 2020 outbreak, various social media outlets published detailed numbers of new confirmed, suspected, and dead cases each day. Disclosure of virus diagnosis and treatment protocols, protection guidelines, and vaccine development information by medical associations as well as the Red Cross Society’s timely counterattack to the rumors led to the rapid recovery of negative social psychology.

**Figure 1:**
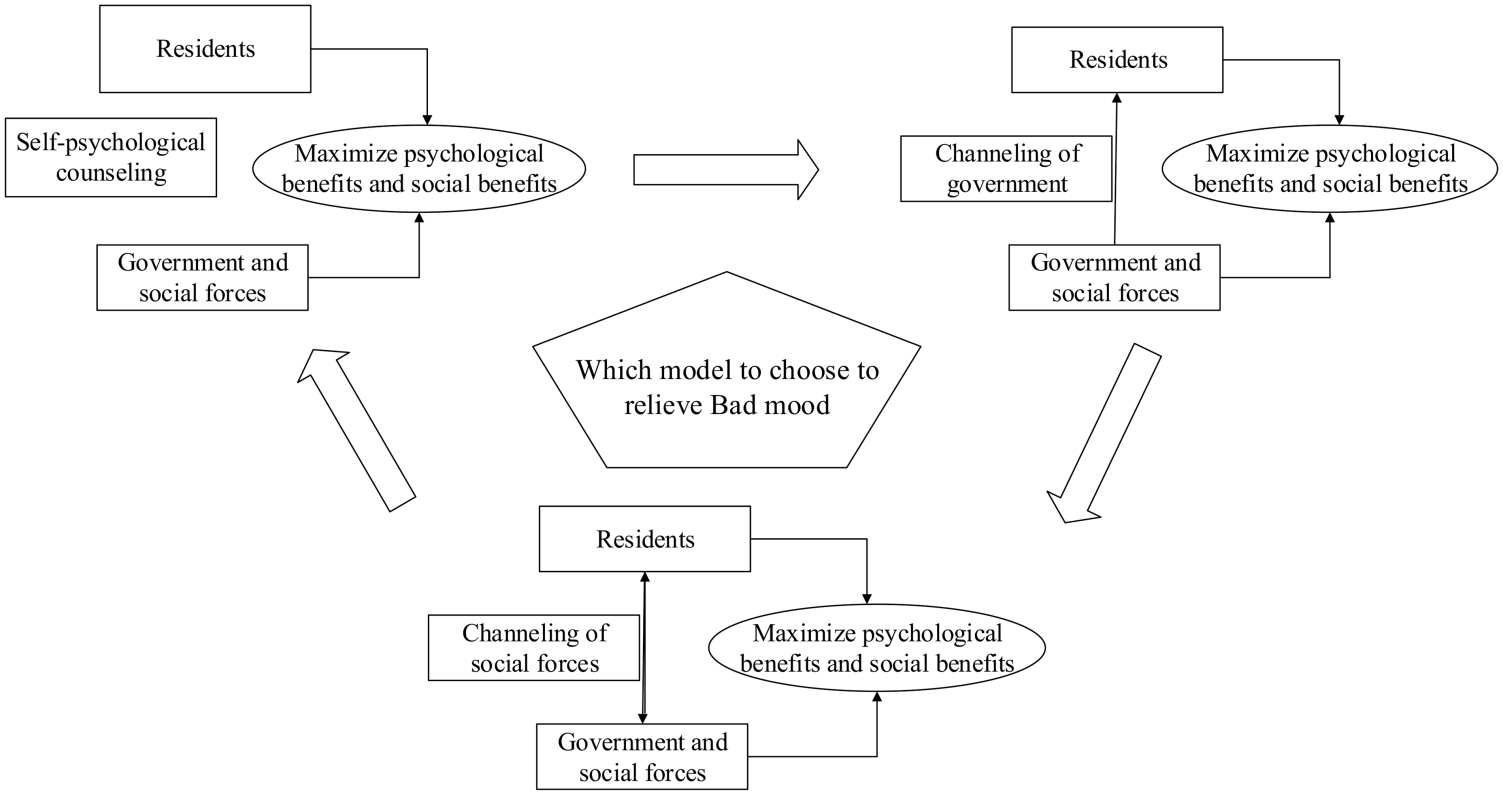
Various modes of psychological counseling for isolated residents.

The relationship between different psychological counseling modes is shown in Figure 1.

### 1.4. Hypothesis

1. Guidance by the government or social forces can weaken the negative psychological adjustment issues. Once the residents are isolated, their psychological fluctuations are more drastic, prone to tension, anxiety, depression, and other conditions. To guide residents to communicate with each other, the government could carry out a free psychological diagnosis for residents, provide mental health screening and psychological counseling services, and assess their recent emotional state (Shen, 2022). Therefore, residents can understand their own emotions and state and pay attention to their mental health level. At the same time, social forces can also be guided to reduce the adverse psychological state of the isolated population. For example, in order to help residents relieve their tension and anxiety and facilitate their lives, the social forces of e-commerce and trade circulation integrate online and offline resources and actively carry out the business of online booking store collection, online booking store delivery, and online booking self-picking (Feng, 2020). This can open up an all-channel, all-weather experience consumption channel.
2. The interaction degree of residents is in a state of continuous dynamic change. After the residents were isolated, the original working and living conditions were greatly affected. At this time, residents can relieve tension, depression, anxiety, and other adverse psychological states through offline and online communication. After the government or social forces guide the residents, it will change the degree of communication among residents. This degree of interaction in turn affects the level of government or social forces to guide. In such a continuous cycle, residents’ degree of communication is in a state of continuous dynamic change.
3. The government adopts a strict “dynamic zero clearance” policy. “Dynamic zeroing” means that once an epidemic occurs in a place, we must fight out together and extinguish it together. When an infection occurs and causes a local case, if we control it very quickly in a very short period of time, the chain of infection is prevented and does not spread continuously, or the chain of infection is wiped out, also known as zeroing out. Once an epidemic occurs, it can be quickly identified and handled to break the chain of transmission, and the community as a whole will gradually move toward dynamic elimination. If dynamic clearing is not pursued, social communication will continue to connect and form a scale rebound. To achieve “dynamic zeroing,” local authorities often quarantine close contacts.

### 1.5. Variable definition

See Table 1.

**Table 1:** The main definition of variables and parameters in this article.

variables and parameters	Specific meaning
*Y =* {*F*, *G*, *S*}	Three modes of psychological counseling under the epidemic situation (self-counseling, government counseling, and social forces counseling)
*Independent variable*	
*F*_*Y*1_(*t*)	The offline communication degree of the public under the mode *Y*
*G*_*Y*1_(*t*)	The degree of public online communication in mode *Y*
*S*_*Y*2_(*t*)	The government’s anti-epidemic efforts in the mode *Y*
*x*_*Y*1_(*t*)	Public enthusiasm for communication under psychological counseling model *Y*
*x*_*Y*2_(*t*)	The reputation of the government in psychotherapy mode *Y*
*Parameter*	
*ρ*	The discount rate that occurs over time, which is the discount factor, 0 ≤ *ρ* ≤ 1
*δ*_1_	The decay rate of enthusiasm, *δ*_1_ > 0
*δ*_2_	The decay rate of the government’s reputation, *δ*_2_ > 0
*b*_on_	Gain from every online interaction, *b*_on_ > 0
*b*_off_	Unit offline contacts to gain income, *b*_off_ > 0
*l*_1_	The positive influence of unit interaction enthusiasm on public psychology, *l*_1_ > 0
*l*_2_	The positive effects of unit reputation, *l*_2_ > 0
*c*_*Y*1_	Risk of infectious diseases in unit offline contacts, *c*_*Y*1_ > 0
*c*_*Y*2_	The cost of government unit effort, *c*_*Y*2_ > 0
*β*_1_	The positive influence of government guidance on communication, *β*_1_ > 0
*d*	The cost of unit government channeling, *d* > 0
*k*	The effect of offline interaction on mood, *k* > 0
*β*_2_	The positive influence of social force guidance on the degree of interaction, 0 < *β*_2_ < *β*_1_
*λ*	The reputation of the government’s units for fighting the pandemic, *λ* > 0
*Function*	
*J*_*Y*1_(*t*)	Benefit function of public psychological satisfaction degree under psychological counseling mode *Y*
*V*_*Y*1_(*t*)	Psychological benefits of the public under psychological counseling model *Y*
*J*_*Y*2_(*t*)	Social welfare function of government under psychological counseling model *Y*
*V*_*Y*2_(*t*)	The social benefits of the government under the psychological counseling model *Y*

## 2. Methodology

The article is original research. It focuses on how to ensure the mental health of isolated people in China under the “dynamic clearance” of patients with COVID-19. In the context of the global COVID-19 pandemic, the psychological state of residents is easily affected by lockdowns, quarantines, and other factors. To describe the whole process of mental state which changes from time to time, a differential game is used in this article. After residents suffer from lockdown or quarantine, timely guidance from the government and social forces can help local residents face the epidemic with a better attitude, thus reducing the negative psychological impact of the epidemic. Government guidance will produce an immediate effect, but to guide the masses, one needs to pay a certain financial cost, further increasing the financial pressure. Although the social force guidance takes a longer time to effect, it will gain the corresponding reputation at the same time. In the process of psychological counseling of the isolated people, no matter the government or social forces, while paying some costs, they can also reap certain benefits.

### 2.1. Differential game

Game theory is concerned with the interaction between formulaic incentive structures (Guo and Harmati, 2022). It is a mathematical theory and method to study the phenomena with the nature of struggle or competition (Xu et al., 2022). In this article, game theory considers the behavior of the government in the game and the individual behavior of the isolated person and studies their optimization strategies. A differential game refers to a time-continuous game played by multiple players in a time-continuous system. It has the goal of optimizing the independence and conflict of each player and can finally obtain the strategy of each player evolving over time and reaching the Nash equilibrium. The theory of differential game originated from research on the pursuit of flight by both sides in the military confrontation carried out by the US Air Force in the 1950s. It is a combination of optimal control and game theory.

In this article, a differential game is used for analysis. First, this article obtains the social income function of the public and the government. Second, changes in public and government state variables are represented. Third, the HJB equation is obtained according to the social income function. Fourth, this article calculates the HJB equation and obtains the optimal control variables and social benefits. Among them, differential games mainly solve the problem of the maximum value in a constantly changing state. There are benefit functions and state variables in this game. HJB is a partial differential equation that is central to optimal control. The solution of the HJB equation is a real-value function with minimum cost under a specific dynamic system and related cost function. Many scholars use the HJB equation to solve differential game problems (Elliott and Siu, 2009; Ma et al., 2015; Lindensj, 2019).

### 2.2. Differential game of different psychological counseling modes

#### 2.2.1. Self-psychological counseling

In the context of “dynamic zeroing,” when an epidemic occurs in a region, the Chinese government quarantines close contacts to prevent the spread of the epidemic. In some cases, the resources at the disposal of governments are limited. The government can only focus on the spread of the disease. At this time, mental health problems in the isolated population cannot be addressed. If the quarantined people have psychological problems, they can only undergo self-counseling.

The social welfare function of the public consists of the benefits gained from online communication, the benefits gained from offline communication, the depression generated by online communication, and the long-term effects of public enthusiasm for communication. The government’s social welfare function consists of the cost of responding to the pandemic and the reputation the government gains. Under the mode of self-psychological counseling, the expression of the public’s psychological benefits and the government’s social welfare function are, respectively,


(1) $$\begin{array}{l} J_{F1}=\overset{\infty }{\underset{0}{\int }}[b_{on}G_{F1}\left(t\right)+b_{off}F_{F1}\left(t\right)-\frac{c_{F1}}{2}G_{F1}^{2}\left(t\right)+\\ \;\;\;\;\;\;\;\;\;\;\;\;\;\;l_{1}x_{F1}\left(t\right)]\;\;e^{-\rho t}dt \end{array}$$

(2) $$J_{F2}=\overset{\infty }{\underset{0}{\int }}[-\frac{c_{F2}}{2}S_{F2}^{2}\left(t\right)+l_{2}x_{F2}\left(t\right)]\;\;e^{-\rho t}dt$$

In (1), *b_on_G*_*F*1_(*t*) represents the benefits obtained from the public’s online communication. *b_off_F*_*F*1_(*t*) represents the income obtained by the public’s offline communication. $\frac{c_{F}}{2}G_{F1}^{2}\left(t\right)$ indicates the level of public depression caused by online interactions. $l_{1}x_{F1}\left(t\right)$ represents the influence of public enthusiasm on psychological benefits. In (2), $\frac{c_{F2}}{2}S_{F2}^{2}\left(t\right)$ represents the cost of the government’s response to the epidemic. $l_{2}x_{F2}\left(t\right)$ represents the impact of government reputation on social benefits.

Changes in public enthusiasm for communication and government reputation under the self-psychological counseling mode are as follows:


(3) $${\dot{x}}_{F1}\left(t\right)=-\lambda F_{F1}^{2}\left(t\right)+\delta x_{F1}\left(t\right)$$

(4) $${\dot{x}}_{F2}\left(t\right)=\lambda S_{F2}\left(t\right)-\delta x_{F2}\left(t\right)$$

In (3) and (4), $-\lambda F_{F1}^{2}\left(t\right)$ indicates the negative influence of offline communication on communication enthusiasm under the mode of public self-psychological counseling. $\delta x_{F1}\left(t\right)$ represents the recovery degree of public enthusiasm for communication under the mode of public self-psychological counseling. $\lambda S_{F2}\left(t\right)$ represents the positive impact of the government’s anti-epidemic efforts on the government’s reputation under the mode of public self-psychological counseling. $\delta x_{F2}\left(t\right)$ represents the decline of the government’s reputation under the mode of public self-psychological counseling.

#### 2.2.2. Psychological counseling by the government

In the mode of government psychological counseling, the government should take the lead and establish a good social communication mechanism. The government has improved the social psychological support system so that the public can obtain psychological support from it. In this mode, it can enhance the public’s recognition of the government and build a government with credibility.

There are some differences between the social welfare function of the government psychological counseling model and the self-psychological counseling model. For example, under the psychological counseling mode of the government, the cost of the government’s response to the epidemic has increased, and the public’s psychological condition has been alleviated to some extent. Under the government psychological counseling model, the expression of the public’s psychological benefits and the government’s social welfare function is as follows:


(5) $$\begin{array}{l} J_{G1}=\overset{\infty }{\underset{0}{\int }}[b_{on}G_{G1}\left(t\right)+b_{off}F_{G1}\left(t\right)-\frac{c_{G1}}{2}G_{G1}^{2}\left(t\right)+\\ \;\;\;\;\;\;\;\;\;\;\;\;\;\;G_{G1}\left(t\right)\ln\left(1+\beta_{1}\right)+l_{1}x_{G1}\left(t\right)]\;\;e^{-\rho t}dt \end{array}$$

(6) $$J_{G2}=\overset{\infty }{\underset{0}{\int }}[-\frac{c_{G2}}{2}{\left(S_{G2}\left(t\right)+d\beta_{1}\right)}^{2}+l_{2}x_{G2}\left(t\right)]\;\;e^{-\rho t}dt$$

In (5) and (6), *b_on_G*_*G*1_(*t*) represents the benefits obtained by the public’s online communication. *b_off_F*_*G*1_(*t*) represents the income obtained by the public’s offline communication. $\frac{c_{F}}{2}G_{G1}^{2}\left(t\right)$ indicates the level of public depression caused by online interactions. $l_{1}x_{G1}\left(t\right)$ represents the influence of public enthusiasm on psychological benefits. $G_{G1}\left(t\right)\ln\left(1+\beta_{1}\right)$ shows the favorable influence of government guidance on communication. $\frac{c_{G2}}{2}{\left(S_{G2}\left(t\right)+d\beta_{1}\right)}^{2}$ represents the cost of the government’s response to the pandemic. $l_{2}x_{G2}\left(t\right)$ represents the impact of government reputation on social benefits.

Under the psychological counseling mode of the government, the public’s communication enthusiasm and the change in the government’s reputation are as follows:


(7) $${\dot{x}}_{G1}\left(t\right)=\left(-\lambda +k\beta_{1}\right)F_{G1}^{2}\left(t\right)+\delta x_{G1}\left(t\right)$$

(8) $${\dot{x}}_{G2}\left(t\right)=\left(\lambda +k\beta_{1}\right)S_{G2}\left(t\right)-\delta x_{G2}\left(t\right)$$

In (7) and (8), $\left(-\lambda +k\beta_{1}\right)F_{G1}^{2}\left(t\right)$ indicates the negative influence of offline communication on communication enthusiasm under the psychological counseling mode of the government. $\delta x_{G1}\left(t\right)$ indicates the recovery degree of public enthusiasm under the psychological counseling mode of the government. $\left(\lambda +k\beta_{1}\right)S_{G2}\left(t\right)$ indicates the positive impact of the government’s anti-epidemic efforts on the government’s reputation under the psychological counseling mode. $\delta x_{G2}\left(t\right)$ represents the decay of the government’s reputation under the psychological counseling mode of government.

#### 2.2.3. Psychological counseling by the social forces

Compared with the first two models, the social power psychological counseling model has some differences in its social welfare function. Under the mode of channeling social forces, the cost of government response to the epidemic remains unchanged. At the same time, this model can also alleviate the psychological problems of the public. Under the social forces’ psychological counseling model, the expression of the public’s psychological benefits and the government’s social welfare function is as follows:


(9) $$\begin{array}{l} J_{S1}=\overset{\infty }{\underset{0}{\int }}[b_{on}G_{S1}\left(t\right)+b_{off}F_{S1}\left(t\right)-\frac{c_{S1}}{2}G_{S1}^{2}\left(t\right)\\ \;\;\;\;\;\;\;\;\;\;+G_{S1}\left(t\right)\ln\left(1+\beta_{2}\right)+l_{1}x_{S1}\left(t\right)]\;\;e^{-\rho t}dt \end{array}$$

(10) $$J_{S2}=\overset{\infty }{\underset{0}{\int }}[-\frac{c_{S2}}{2}S_{S2}{\left(t\right)}^{2}+l_{2}x_{S2}\left(t\right)]\;\;e^{-\rho t}dt$$

In (9), *b_on_G*_*S*1_(*t*) represents the benefits obtained from the public’s online communication. *b_off_F*_*S*1_(*t*) represents the income obtained by the public offline communication. $\frac{c_{S1}}{2}G_{S1}^{2}\left(t\right)$ indicates the level of public depression caused by online interactions. $l_{1}x_{S1}\left(t\right)$ represents the influence of public enthusiasm on psychological benefits. $G_{S1}\left(t\right)\ln\left(1+\beta_{2}\right)$ represents the favorable influence of social force guidance on communication. In (10), $\frac{c_{S2}}{2}S_{S2}{\left(t\right)}^{2}$ represents the cost of the government’s response to the epidemic. $l_{2}x_{S2}\left(t\right)$ represents the impact of government reputation on social benefits.

Under the mode of channeling social forces, the changes in public enthusiasm and government reputation can be expressed as follows:


(11) $${\dot{x}}_{S1}\left(t\right)=\left(-\lambda +k\beta_{2}\right)F_{S1}^{2}\left(t\right)+\delta x_{S1}\left(t\right)$$

(12) $${\dot{x}}_{S2}\left(t\right)=\left(\lambda +k\beta_{2}\right)S_{S2}\left(t\right)-\delta x_{S2}\left(t\right)$$

In (11) and (12), $\left(-\lambda +k\beta_{2}\right)F_{S1}^{2}\left(t\right)$ represents the negative influence of offline communication on the enthusiasm of communication under the mode of social power psychological counseling. $\delta x_{S1}\left(t\right)$ represents the recovery degree of public enthusiasm for communication under the psychological counseling mode of social forces. $\left(\lambda +k\beta_{2}\right)S_{S2}\left(t\right)$ represents the positive impact of the government’s anti-epidemic efforts on the government’s reputation under the psychological counseling mode of social forces. $\delta x_{S2}\left(t\right)$ represents the decay of the government’s reputation under the psychological counseling mode of social forces.

## 3. Results

The social benefits obtained by the government and the psychological benefits of the public are not only affected by the control variables and parameters but also constantly change with the impact of time, state, and state on social welfare. The HJB formula was used in order to better calculate the balanced degree of public interaction, the degree of the government’s anti-epidemic efforts, psychological benefits, and social benefits. The formula is based on dynamic programming developed in the 1950s by Richard Behrman and his colleagues. The HJB formula is a partial differential equation, which is the core of optimal control.

### 3.1. HJB formula

If the public conducts self-psychological counseling, then in the time *t*∈[0,+∞), the HJB formula of the psychological benefit obtained by the public and the government’s social welfare function in this mode is as follows:


(13) $$\begin{array}{l} \rho V_{F1}=\underset{F_{F1}\left(t\right),G_{F1}\left(t\right)}{\max}\{[b_{on}G_{F1}\left(t\right)+b_{off}F_{F1}\left(t\right)-\\ \;\;\;\;\;\;\;\;\;\;\;\;\;\;\;\;\;\frac{c_{F1}}{2}G_{F1}^{2}\left(t\right)+l_{1}x_{F1}\left(t\right)]+\\ \;\;\;\;\;\;\;\;\;\;\;\;\;\;\;\;\;\frac{\partial V_{F1}}{\partial x_{F1}}\left[-\lambda F_{F1}^{2}\left(t\right)+\delta x_{F1}\left(t\right)\right]\} \end{array}$$

(14) $$\begin{array}{l} \rho V_{F2}=\underset{S_{F2}\left(t\right)}{\max}\{[-\frac{c_{F2}}{2}S_{F2}^{2}\left(t\right)+l_{2}x_{F2}\left(t\right)]+\\ \;\;\;\;\;\;\;\;\;\;\;\;\;\;\;\;\;\;\frac{\partial V_{F2}}{\partial x_{F2}}\left[\lambda S_{F2}\left(t\right)-\delta x_{F2}\left(t\right)\right]\} \end{array}$$

If the government conducts psychological counseling, then in the time *t*∈[0,+∞), the HJB formula of the psychological benefit obtained by the public and the government’s social welfare function in this mode is as follows:


(15) $$\begin{array}{l} \rho V_{G1}=\underset{F_{G1}\left(t\right),G_{G1}\left(t\right)}{\max}\{[b_{on}G_{G1}\left(t\right)+b_{off}F_{G1}\left(t\right)-\;\;\\ \begin{array}{l} \;\;\;\;\;\;\;\;\;\;\;\;\;\;\;\;\frac{c_{G1}}{2}G_{G1}^{2}\left(t\right)+G_{G1}\left(t\right)\ln\left(1+\beta_{1}\right)+l_{1}x_{G1}\left(t\right)]\\ \;\;\;\;\;\;\;\;\;\;\;\;\;+\frac{\partial V_{G1}}{\partial x_{G1}}\left[\left(-\lambda +k\beta_{1}\right)F_{G1}^{2}\left(t\right)+\delta x_{G1}\left(t\right)\right]\} \end{array} \end{array}$$

(16) $$\begin{array}{l} \rho V_{G2}=\underset{S_{G2}\left(t\right)}{\max}\{[-\frac{c_{G2}}{2}{\left(S_{G2}\left(t\right)+d\beta_{1}\right)}^{2}+l_{2}x_{G2}\left(t\right)]+\\ \;\;\;\;\;\;\;\;\;\;\;\;\;\;\;\;\;\;\frac{\partial V_{G2}}{\partial x_{G2}}\left[\left(\lambda +k\beta_{1}\right)S_{G2}\left(t\right)-\delta x_{G2}\left(t\right)\right]\} \end{array}$$

If social forces conduct psychological guidance, then in the time *t*∈[0,+∞), the HJB formula of the psychological benefit obtained by the public and the social welfare function of the government in this mode is as follows:


(17) $$\begin{array}{l} \begin{array}{l} \rho V_{S1}=\underset{F_{S1}\left(t\right),G_{S1}\left(t\right)}{\max}\overset{\infty }{\underset{0}{\int }}[b_{on}G_{S1}\left(t\right)+b_{off}F_{S1}\left(t\right)-\\ \;\;\;\;\;\;\;\;\;\;\;\;\;\;\;\;\;\frac{c_{G1}}{2}G_{S1}^{2}\left(t\right)+G_{S1}\left(t\right)\ln\left(1+\beta_{2}\right)+l_{1}x_{S1}\left(t\right)] \end{array}\\ \;\;\;\;\;\;\;\;\;\;\;\;\;\;\;\;\frac{\partial V_{S1}}{\partial x_{S1}}\left[\left(-\lambda +k\beta_{2}\right)F_{S1}^{2}\left(t\right)+\delta x_{S1}\left(t\right)\right]\} \end{array}+$$

(18) $$\begin{array}{l} \rho V_{S2}=\underset{S_{S2}\left(t\right)}{\max}\{[-\frac{c_{G2}}{2}S_{S2}{\left(t\right)}^{2}+l_{2}x_{S2}\left(t\right)]+\\ \;\;\;\;\;\;\;\;\;\;\;\;\;\;\;\;\;\;\frac{\partial V_{S2}}{\partial x_{S2}}\left[\left(\lambda +k\beta_{2}\right)S_{S2}\left(t\right)-\delta x_{S2}\left(t\right)\right]\} \end{array}$$

### 3.2. Result of equilibrium

#### 3.2.1. Self-psychological counseling

Proposition 1: Under the mode of self-psychological counseling, the public’s online communication degree, the public’s offline communication degree, and the government’s anti-epidemic effort degree are, respectively (refer to Supplementary Appendix 1 for the specific solution process):

(19), $$G_{F1}^{*}\left(t\right)=\frac{b_{on}}{c_{F1}},F_{F1}^{*}\left(t\right)=\frac{b_{off}}{2\lambda }\frac{\rho -\delta }{l_{1}}$$

(20) $$S_{F2}^{*}\left(t\right)=\frac{\lambda }{c_{F2}}\left(\frac{l_{2}}{\rho +\delta }\right)$$

The public’s equilibrium psychological benefit and the government’s equilibrium welfare function are as follows:


(21) $$\begin{array}{l} V_{F1}^{*}=\frac{l_{1}}{\rho -\delta }x_{F1}+\frac{1}{\rho }[b_{on}\frac{b_{on}}{c_{F1}}+\frac{{\left(b_{off}\right)}^{2}}{2\lambda }{\left(\frac{l_{1}}{\rho -\delta }\right)}^{-1}-\\ \frac{c_{F1}}{2}{\left(\frac{b_{on}}{c_{F1}}\right)}^{2}]+\frac{1}{\rho }\frac{l_{1}}{\rho -\delta }\left[-\lambda {\left(\frac{b_{off}}{2\lambda }\right)}^{2}{\left(\frac{l_{1}}{\rho -\delta }\right)}^{-2}\right] \end{array}$$

(22) $$\begin{array}{l} V_{F2}^{*}=\frac{l_{2}}{\rho +\delta }x_{F2}-\frac{1}{\rho }\frac{c_{F2}}{2}{\left(\frac{\lambda }{c_{F2}}\right)}^{2}{\left(\frac{l_{2}}{\rho +\delta }\right)}^{2}+\\ \;\;\;\;\;\;\;\;\;\;\;\;\;\;\frac{1}{\rho }\frac{l_{2}}{\rho +\delta }\frac{\lambda^{2}}{c_{F2}}\left(\frac{l_{2}}{\rho +\delta }\right) \end{array}$$

Conclusion 1: Under the self-psychological counseling mode, the offline communication degree of the public is inversely proportional to the positive influence of the unit’s communication enthusiasm on public psychology and the reputation of the government unit’s efforts. The degree of the government’s anti-epidemic efforts is directly proportional to the reputation of the government unit and the positive impact of the unit’s reputation.

#### 3.2.2. Psychological counseling by the government

Proposition 1: Under the mode of psychological counseling by the government, the public’s online communication degree, the public’s offline communication degree, and the government’s anti-epidemic effort degree are, respectively (refer to Supplementary Appendix 2 for the specific solution process):

(23), $$\begin{array}{l} G_{G1}^{*}\left(t\right)=\frac{b_{on}+\ln\left(1+\beta_{1}\right)}{c_{G1}},F_{G1}^{*}\left(t\right)=\\ \;\;\;\;\;\;\;\;\;\;\;\;\;\;\;\;\;\;\;\;\;\frac{-b_{off}}{2\left(-\lambda +k\beta_{1}\right)}{\left(\frac{l_{1}}{\rho -\delta }\right)}^{-1} \end{array}$$

(24) $$S_{G2}^{*}\left(t\right)=-d\beta_{1}+\frac{1}{c_{G2}}\frac{l_{2}}{\rho +\delta }\left(\lambda +k\beta_{1}\right)$$

The public’s equilibrium psychological benefit and the government’s equilibrium welfare function are as follows:


(25) $$\begin{array}{l} V_{G1}^{*}=\frac{l_{1}}{\rho -\delta }x_{G1}+\frac{1}{\rho }[b_{on}\frac{b_{on}+\ln\left(1+\beta_{1}\right)}{c_{G1}}+\\ \begin{array}{l} \;\;\;\;\;\;\;\;\;\;\;\;\frac{-{\left(b_{off}\right)}^{2}}{2\left(-\lambda +k\beta_{1}\right)}{\left(\frac{l_{1}}{\rho -\delta }\right)}^{-1}\;-\frac{c_{G1}}{2}{\left(\frac{b_{on}+\ln\left(1+\beta_{1}\right)}{c_{G1}}\right)}^{2}+ \end{array}\\ \begin{array}{l} \;\;\;\;\;\;\;\;\;\;\;\;\frac{b_{on}+\ln\left(1+\beta_{1}\right)}{c_{G1}}\ln\left(1+\beta_{1}\right)]+\frac{1}{\rho }\frac{l_{1}}{\rho -\delta }\\ \;\;\;\;\;\;\;\;\;\;\;\left[\left(-\lambda +k\beta_{1}\right){\left(\frac{-b_{off}}{2\left(-\lambda +k\beta_{1}\right)}{\left(\frac{l_{1}}{\rho -\delta }\right)}^{-1}\right)}^{2}\right] \end{array} \end{array}$$

(26) $$\begin{array}{l} \begin{array}{l} V_{G2}^{*}=\frac{l_{2}}{\rho +\delta }x_{B2}-\\ \;\;\;\frac{c_{G2}}{2}\frac{1}{\rho }{\left[\frac{1}{c_{G2}}\left(-c_{G2}d\beta_{1}+\frac{l_{2}}{\rho +\delta }\left(\lambda +k\beta_{1}\right)\right)+d\beta_{1}\right]}^{2} \end{array}\\ \;\;+\frac{1}{\rho }\frac{l_{2}}{\rho +\delta }\left[\left(\lambda +k\beta_{1}\right)\frac{1}{c_{G2}}\left(-c_{G2}d\beta_{1}+\frac{l_{2}}{\rho +\delta }\left(\lambda +k\beta_{1}\right)\right)\right] \end{array}$$

Conclusion 2: Compared with the self-psychological counseling of the public, the government’s psychological counseling of the public can increase the degree of online and offline communication of the public.

#### 3.2.3. Psychological counseling by the social forces

Proposition 1: Under the mode of psychological counseling by the social forces, the public’s online communication degree, the public’s offline communication degree, and the government’s anti-epidemic effort degree are, respectively (refer to Supplementary Appendix 3 for the specific solution process):

(27), $$\begin{array}{l} G_{S1}^{*}\left(t\right)=\frac{b_{on}+\ln\left(1+\beta_{2}\right)}{c_{S1}},F_{S1}^{*}\left(t\right)=\\ \;\;\;\;\;\;\;\;\;\;\;\;\;\;\;\;\;\;\;\;\frac{-b_{off}}{2\left(-\lambda +k\beta_{2}\right)}{\left(\frac{l_{1}}{\rho -\delta }\right)}^{-1} \end{array}$$

(28) $$S_{S2}^{*}\left(t\right)=\frac{\lambda +k\beta_{2}}{c_{S2}}\frac{l_{2}}{\rho +\delta }$$

The public’s equilibrium psychological benefit and the government’s equilibrium welfare function are as follows:


(29) $$\begin{array}{l} V_{S1}^{*}=\frac{l_{1}}{\rho -\delta }x_{S1}+\frac{1}{\rho }[b_{on}\frac{b_{on}+\ln\left(1+\beta_{2}\right)}{c_{S1}}+\\ \begin{array}{l} \;\;\;\;\;\;\;b_{off}\frac{-b_{off}}{2\left(-\lambda +k\beta_{2}\right)}{\left(\frac{l_{1}}{\rho -\delta }\right)}^{-1}-\frac{c_{G1}}{2}{\left(\frac{b_{on}+\ln\left(1+\beta_{2}\right)}{c_{S1}}\right)}^{2}+\\ \;\;\;\;\;\;\;\frac{b_{on}+\ln\left(1+\beta_{2}\right)}{c_{S1}}\ln\left(1+\beta_{2}\right)]+\\ \;\;\;\;\;\;\;\;\left[\left(-\lambda +k\beta_{2}\right){\left(\frac{-b_{off}}{2\left(-\lambda +k\beta_{2}\right)}\right)}^{2}{\left(\frac{l_{1}}{\rho -\delta }\right)}^{-2}\right]\frac{l_{1}}{\rho -\delta }\frac{1}{\rho }\;\; \end{array} \end{array}$$

(30) $$\begin{array}{l} V_{S2}^{*}=\frac{l_{2}}{\rho +\delta }x_{S2}-\frac{1}{\rho }\frac{c_{S2}}{2}{\left(\frac{\lambda +k\beta_{2}}{c_{S2}}\frac{l_{2}}{\rho +\delta }\right)}^{2}+\\ \;\;\;\;\;\;\;\;\;\;\;\;\;\;{\left(\frac{l_{2}}{\rho +\delta }\right)}^{2}\frac{1}{\rho }\left(\lambda +k\beta_{2}\right)\frac{\lambda +k\beta_{2}}{c_{S2}}\;\; \end{array}$$

Conclusion 3: Psychological counseling of social power can increase the degree of public communication. If the degree of the government’s guidance is the same as that of social forces, then the degree of the government’s anti-epidemic efforts under the mode of social forces’ guidance is greater than that under the mode of government guidance.

### 3.3. Comparison of equilibrium results

To better compare the psychological benefits and social benefits of different psychological counseling modes, this article calculates the difference between them.

Through calculation, the difference in psychological benefits between the government psychological counseling mode and the self-counseling mode is as follows:


(31) $$\begin{array}{l} V_{G1}^{*}-V_{F1}^{*}=\frac{1}{\rho }[\frac{-{\left(b_{off}\right)}^{2}}{4\left(-\lambda +k\beta_{1}\right)}{\left(\frac{l_{1}}{\rho -\delta }\right)}^{-1}+\\ \;\;\;\;\;\;\;\;\;\;\;\;\;\;\;\;\;\;\;\;\;\;\;\;\;\;\;\;\;\frac{c_{G1}}{2}{\left(\frac{b_{on}+\ln\left(1+\beta_{1}\right)}{c_{G1}}\right)}^{2}]\;\;-\\ \;\;\;\;\;\;\;\;\;\;\;\;\;\;\;\;\;\;\;\;\;\;\;\;\;\;\;\frac{1}{\rho }[\frac{1}{2}b_{on}\frac{b_{on}}{c_{F1}}+\frac{{\left(b_{off}\right)}^{2}}{4\lambda }{\left(\frac{l_{1}}{\rho -\delta }\right)}^{-1}] \end{array}$$

Through calculation in this article, the difference in social benefits between the government psychological counseling mode and the self-counseling mode is as follows:


(32) $$\begin{array}{l} V_{G2}^{*}-V_{F2}^{*}=-\frac{c_{G2}}{2}\frac{1}{\rho }\\ \begin{array}{l} {\left[\frac{1}{c_{G2}}\left(-c_{G2}d\beta_{1}+\frac{l_{2}}{\rho +\delta }\left(\lambda +k\beta_{1}\right)\right)+d\beta_{1}\right]}^{2}+\\ \frac{1}{\rho }\frac{l_{2}}{\rho +\delta }\left[\left(\lambda +k\beta_{1}\right)\frac{1}{c_{G2}}\left(-c_{G2}d\beta_{1}+\frac{l_{2}}{\rho +\delta }\left(\lambda +k\beta_{1}\right)\right)\right] \end{array}\\ -\frac{1}{2\rho }\frac{l_{2}}{\rho +\delta }\frac{\lambda^{2}}{c_{F2}}\left(\frac{l_{2}}{\rho +\delta }\right) \end{array}$$

Through calculation in this article, the difference in psychological benefits between the social power psychological guidance model and the self-guidance model is as follows:


(33) $$\begin{array}{l} \begin{array}{l} V_{S1}^{*}-V_{F1}^{*}=\frac{1}{\rho }[\frac{c_{G1}}{2}{\left(\frac{b_{on}+\ln\left(1+\beta_{2}\right)}{c_{S1}}\right)}^{2}+\\ \;\;\;\;\;\;\;\;\;\;\;\;\;\;\;\;\;\;\;\;\;\;\;\;\;\;\;b_{off}\frac{-b_{off}}{4\left(-\lambda +k\beta_{2}\right)}{\left(\frac{l_{1}}{\rho -\delta }\right)}^{-1}] \end{array}\\ \;\;\;\;\;\;\;\;\;\;\;\;\;\;-\frac{1}{\rho }[\frac{c_{F1}}{2}{\left(\frac{b_{on}}{c_{F1}}\right)}^{2}+\frac{{\left(b_{off}\right)}^{2}}{4\lambda }{\left(\frac{l_{1}}{\rho -\delta }\right)}^{-1}]\;\; \end{array}$$

After calculation in this article, the difference between the social power psychological guidance model and the self-guidance model of social benefits is as follows:


(34) $$\begin{array}{l} V_{S2}^{*}-V_{F2}^{*}=-\frac{1}{\rho }\frac{c_{S2}}{2}{\left(\frac{\lambda +k\beta_{2}}{c_{S2}}\frac{l_{2}}{\rho +\delta }\right)}^{2}+{\left(\frac{l_{2}}{\rho +\delta }\right)}^{2}\\ \;\;\;\;\;\;\;\frac{1}{\rho }\left(\lambda +k\beta_{2}\right)\frac{\lambda +k\beta_{2}}{c_{S2}}-\frac{1}{\rho }\frac{c_{F2}}{2}{\left(\frac{\lambda }{c_{F2}}\right)}^{2}{\left(\frac{l_{2}}{\rho +\delta }\right)}^{2}\;\; \end{array}$$

Through calculation, the difference in psychological benefits between the psychological counseling model of social forces and the psychological counseling model of the government is as follows:


(35) $$\begin{array}{l} \begin{array}{l} V_{G1}^{*}-V_{S1}^{*}=\frac{1}{\rho }[\frac{-{\left(b_{off}\right)}^{2}}{4\left(-\lambda +k\beta_{1}\right)}{\left(\frac{l_{1}}{\rho -\delta }\right)}^{-1}+\frac{c_{G1}}{2}{\left(\frac{b_{on}+\ln\left(1+\beta_{1}\right)}{c_{G1}}\right)}^{2}]\;\;\\ -\frac{1}{\rho }[\frac{1}{2}b_{on}\frac{b_{on}}{c_{F1}}+\frac{{\left(b_{off}\right)}^{2}}{4\lambda }{\left(\frac{l_{1}}{\rho -\delta }\right)}^{-1}] \end{array}\\ -\frac{1}{\rho }[\frac{c_{G1}}{2}{\left(\frac{b_{on}+\ln\left(1+\beta_{2}\right)}{c_{S1}}\right)}^{2}+\;b_{off}\frac{-b_{off}}{4\left(-\lambda +k\beta_{2}\right)}{\left(\frac{l_{1}}{\rho -\delta }\right)}^{-1}]\\ +\frac{1}{\rho }[\frac{c_{F1}}{2}{\left(\frac{b_{on}}{c_{F1}}\right)}^{2}+\frac{{\left(b_{off}\right)}^{2}}{4\lambda }{\left(\frac{l_{1}}{\rho -\delta }\right)}^{-1}]\;\; \end{array}$$

According to the calculation in this article, the difference in social benefits between the psychological counseling model of social forces and the psychological counseling model of the government is as follows:


(36) $$\begin{array}{l} V_{G2}^{*}-V_{S2}^{*}=-\frac{c_{G2}}{2}\frac{1}{\rho }{\left[\frac{1}{c_{G2}}\left(-c_{G2}d\beta_{1}+\frac{l_{2}}{\rho +\delta }\left(\lambda +k\beta_{1}\right)\right)+d\beta_{1}\right]}^{2}+\\ \;\;\;\frac{1}{\rho }\frac{l_{2}}{\rho +\delta }\left[\left(\lambda +k\beta_{1}\right)\frac{1}{c_{G2}}\left(-c_{G2}d\beta_{1}+\frac{l_{2}}{\rho +\delta }\left(\lambda +k\beta_{1}\right)\right)\right]-\\ \;\;\;\;\frac{1}{2\rho }\frac{l_{2}}{\rho +\delta }\frac{\lambda^{2}}{c_{F2}}\left(\frac{l_{2}}{\rho +\delta }\right)\;+\frac{1}{\rho }\frac{c_{S2}}{2}{\left(\frac{\lambda +k\beta_{2}}{c_{S2}}\frac{l_{2}}{\rho +\delta }\right)}^{2}-{\left(\frac{l_{2}}{\rho +\delta }\right)}^{2}\;\\ \;\;\;\frac{1}{\rho }\left(\lambda +k\beta_{2}\right)\frac{\lambda +k\beta_{2}}{c_{S2}}+\frac{1}{\rho }\frac{c_{F2}}{2}{\left(\frac{\lambda }{c_{F2}}\right)}^{2}{\left(\frac{l_{2}}{\rho +\delta }\right)}^{2}\; \end{array}$$

This article sets specific values for the abovementioned parameters according to the actual situation. For example, the discount factor *ρ* is 0.9. The decay rate *δ*_1_ of enthusiasm is 0.1. The decay rate *δ*_2_ of government reputation is 0.1. The revenue *b*_on_ from the unit-line interaction is 3. *b*_off_ of unit offline communication is 5. The positive influence *l*_1_ of unit interaction enthusiasm on public psychology is 2. The positive impact *l*_2_ of unit reputation l2 is 1.5. The cost *d* per unit of government channeling is 1. The risk of infection *c*_*Y*1_ is 2. The cost *c*_*Y*2_ of the government unit’s effort level is 3. The reputation *λ* of government units for their efforts is 1. The influence *k* of the increase in offline communication on communication is 1.

Therefore, this article can calculate:


(37) $$V_{G1}^{*}-V_{F1}^{*}=2.78\times \frac{1}{\beta_{1}-1}+2.22\ln\left(1+\beta_{1}\right)-2.78$$

(38) $$V_{G2}^{*}-V_{F2}^{*}=-1.25\beta_{1}^{2}-0.834\beta_{1}$$

**Figure 2:**
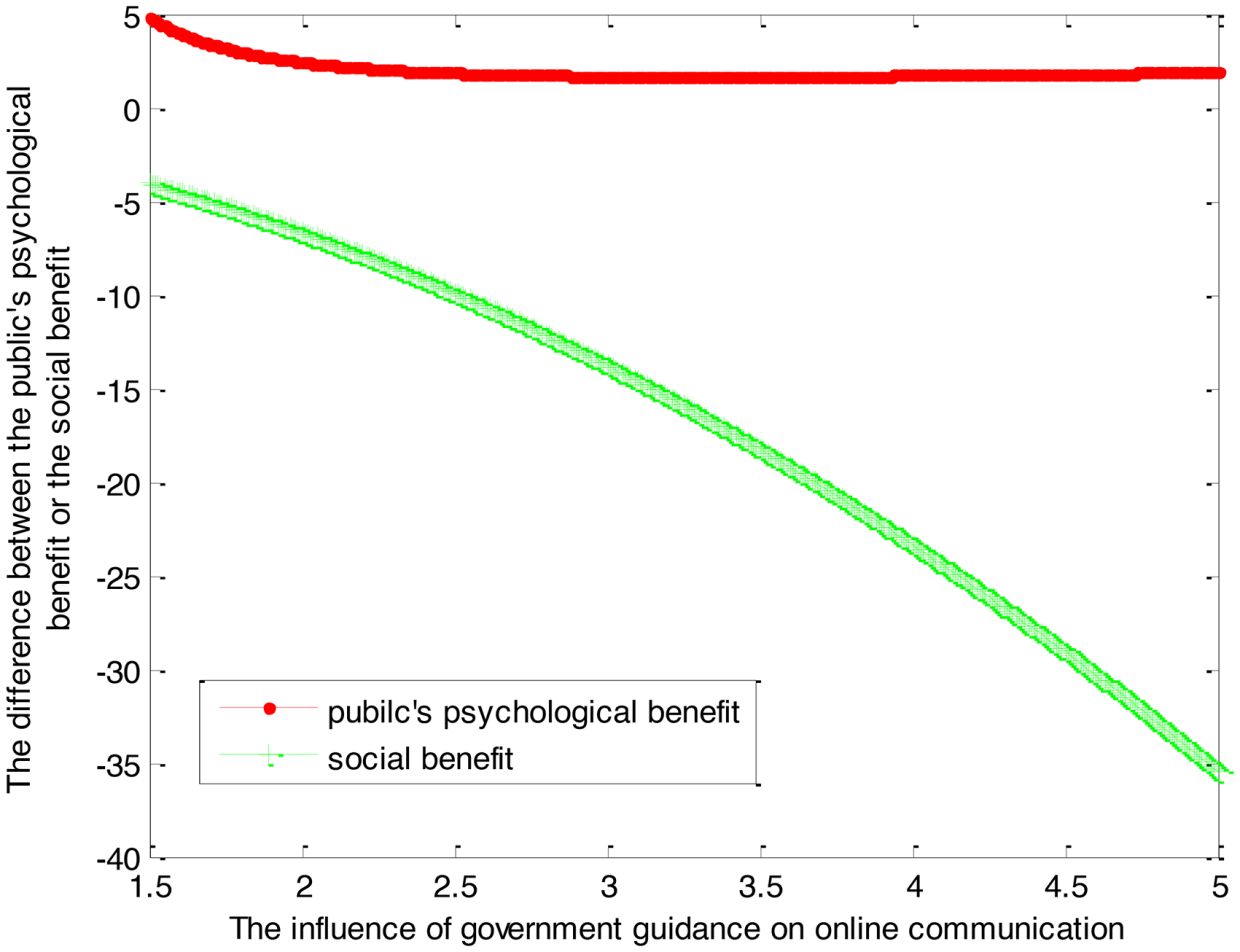
The difference between self-guidance and government guidance.

This article can make the graph as shown in Figure 2.

Conclusion 4: Compared with the self-counseling mode, the public under the government counseling mode can obtain more psychological benefits. However, with the increase of government guidance, the difference between the psychological benefits obtained by self-guidance and the psychological benefits obtained by government guidance becomes smaller at first and then becomes stable. Under the psychological counseling mode of the government, the social benefits of the government decrease, and the more the government counseling, the smaller the social benefits.

(39) $$V_{S2}^{*}-V_{F2}^{*}=0.42\beta_{2}^{2}+0.833\beta_{2}$$

(40) $$V_{S1}^{*}-V_{F1}^{*}=2.22\ln\left(1+\beta_{2}\right)-2.78\times \frac{1}{\beta_{2}-1}-2.78\;\;$$

**Figure 3:**
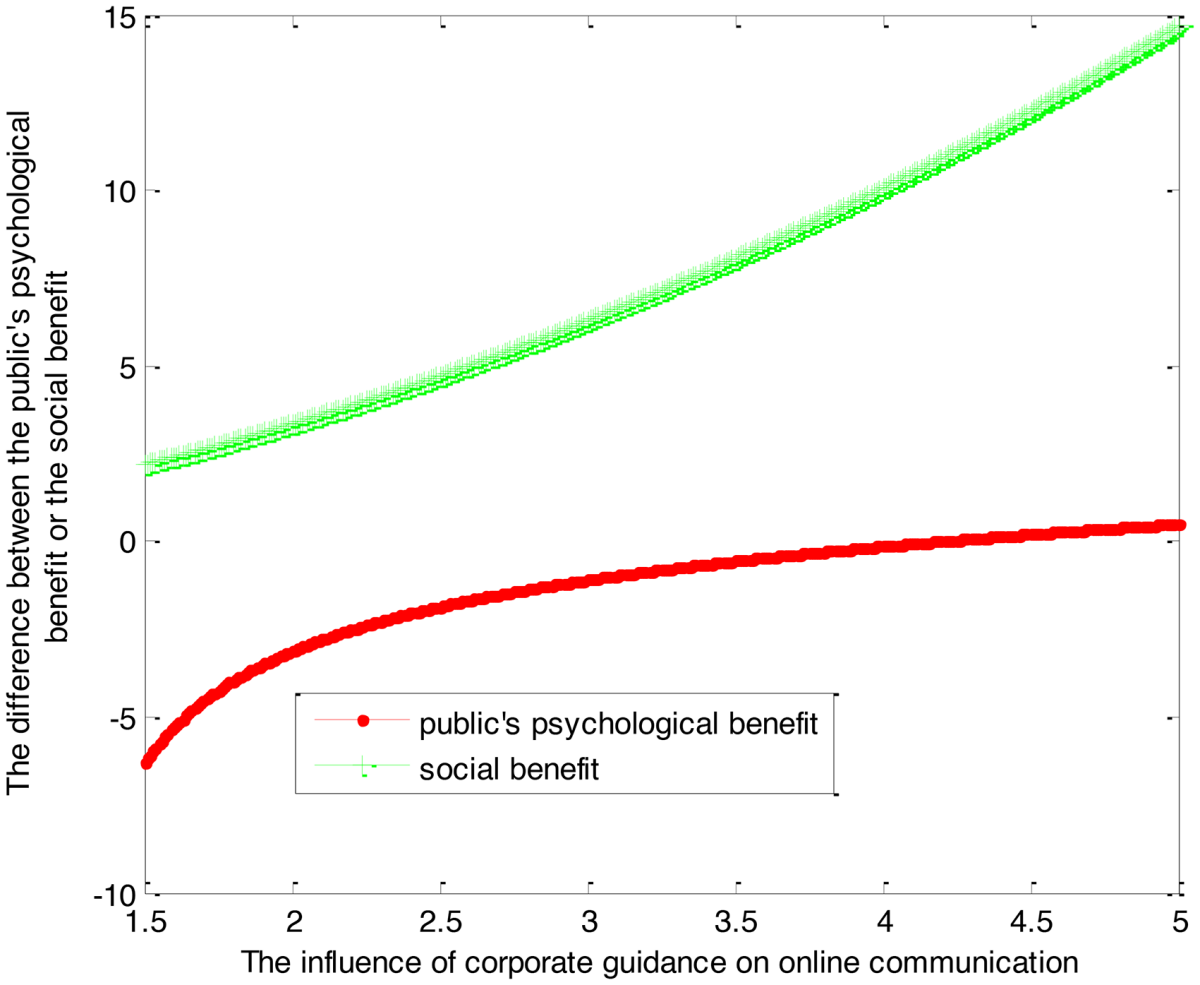
The difference between self-guidance and social force guidance.

This article can make the graph as shown in Figure 3.

Conclusion 5: When the intensity of social power channeling is small, the psychological benefits of the public are smaller than the psychological benefits of self-regulation. With the increase of social forces, the public’s psychological benefits gradually become larger. Ultimately, it will be greater than the psychological benefits of self-counseling. The government’s social benefits gradually increase when the social forces are channeled gradually.

(41) $$\begin{array}{l} V_{G1}^{*}-V_{S1}^{*}=2.78\times \frac{1}{\beta_{1}-1}+2.22\ln\left(1+\beta_{1}\right)-\\ \;\;\;\;\;\;\;\;\;\;\;\;\;\;\;\;\;\;\;\;\;\;\;\;\;\;\;2.22\ln\left(1+\beta_{2}\right)+2.78\times \frac{1}{\beta_{2}-1} \end{array}$$

(42) $$V_{G2}^{*}-V_{S2}^{*}=-1.25\beta_{1}^{2}-0.834\beta_{1}-0.42\beta_{2}^{2}-0.833\beta_{2}$$

**Figure 4:**
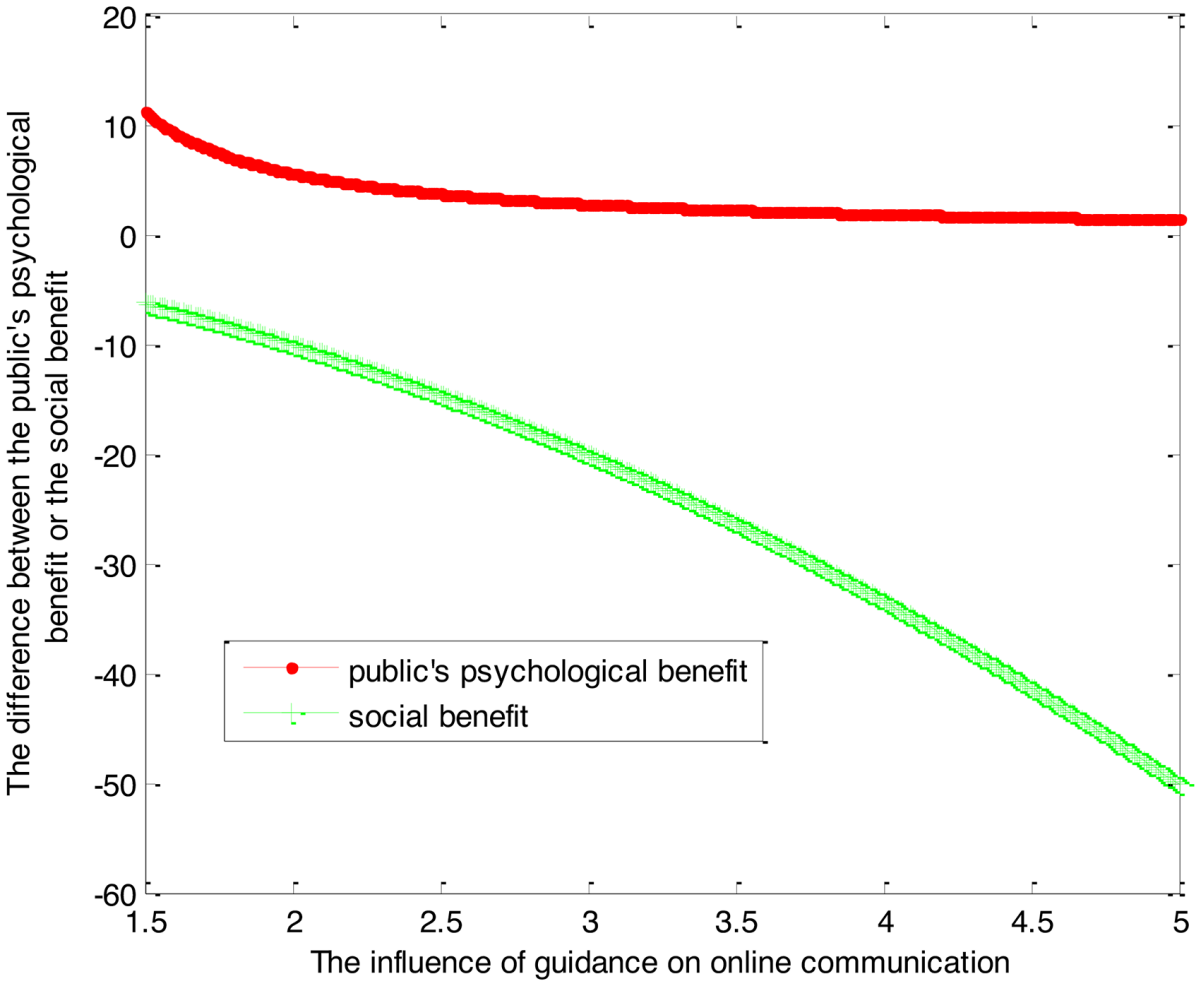
The difference between government guidance and social force guidance.

This article can make the graph as shown in Figure 4.

Conclusion 6: Compared with psychological counseling of social forces, the public under the government’s psychological counseling mode can obtain more psychological benefits. However, with the increase in the government’s efforts to dredge, the difference between the psychological benefits obtained by the government and the psychological benefits obtained by the social forces to dredge decreases at first and then becomes stable. In psychological counseling mode, the social benefits of the government decrease, and the more the counseling, the smaller the social benefits.

## 4. Discussion

As in economic activity and daily life, residents are mobile. This could easily lead to the spread of the epidemic. After an outbreak in a region of China, the government quarantined the local population. This will cause anxiety, tension, depression, and other psychological problems. Considering that the government and social forces can actively guide the local people to alleviate the abovementioned psychological problems. Although the government’s psychological counseling model can produce immediate results, it is easy to affect local financial pressure. Although the mode of social power channeling has a slower effect, it will not have a negative impact on local finance because social power can gain benefits. Therefore, the choice of guided communication mode is an important issue in this study. Since most of the existing literature analyzes the impact of COVID-19, the impact of isolation, and how to mitigate these adverse effects, it has not been found to conduct research from the perspective of guided communication. In particular, it has not been found that guidance communication is divided into two modes, namely, government guidance and social force guidance. In this study, game theory is applied to solve psychological problems caused by the novel coronavirus epidemic, especially considering how the government and social forces achieve optimal psychological and social benefits under different ways of guidance and communication.

This article constructs the differential game model of self-psychological counseling, government psychological counseling, and social force psychological counseling; obtains the psychological benefits of the masses and the benefits of the whole society under the three modes; and compares the applicable conditions of various connection modes. The contribution of this article is as follows: first, this article studies how to provide psychological guidance to the isolated population and divides the guidance into government psychological guidance and social psychological guidance. Second, this article proposes three models to alleviate the impact of the epidemic and quarantine, namely, self-counseling, government counseling, and social forces counseling. Third, this article obtains the psychological benefits of the public and the social benefits of the government under various modes and makes a numerical analysis of them. Finally, this article obtains the applicable scope of each mode, which provides a reference for choosing which mode to conduct psychological counseling.

When infected persons and close contacts are quarantined, in order to prevent their psychological problems, the government should actively provide them with psychological counseling. This is because both community isolation and centralized isolation will have an impact on the mental health of those who are isolated (Ju et al., 2021). For example, after the outbreak of COVID-19, the government will rely on the renovation and construction of exhibition centers and sports stadiums to build makeshift hospitals for the centralized treatment of patients with mild COVID-19. Compared with other traditional hospitals with several patients in one room, makeshift hospitals have effectively alleviated the “difficulty in receiving and receiving” COVID-19 patients, enabling patients with mild COVID-19 to receive timely and effective treatment. Makeshift hospitals are equipped with a large number of medical staff, which can not only treat patients with COVID-19 physically but also effectively relieve their negative psychological conditions. Despite the temporary isolation, the “little joy” of the people in the makeshift hospitals never disappeared under the care of the medical staff, and there was no lack of laughter in the ward. As makeshift hospitals can enable patients to communicate effectively and even do some collective activities, patients feel comfortable (Zhang, 2020).

The purpose of social psychological counseling Is to ensure that people’s right to know and related needs are constantly satisfied, so as to reduce the accumulation of negative psychology. However, when the intensity of such social support is small, the right to know about isolated people will not be satisfied, which will further aggravate the negative psychology of isolated people. Only by increasing the psychological counseling of social forces to the quarantined people, they can relieve their bad emotions such as tension, anxiety, and depression. Both the government and social forces should conduct psychological counseling for the masses. In addition, social forces have helped the government focus on fighting the epidemic. The government has adhered to the general policy of “dynamic zero elimination” intensified efforts to strengthen key epidemic prevention and control work, and strictly implemented measures such as inspection, health monitoring, and personnel management, which have played a crucial role in epidemic prevention and control (Burki, 2022). At this point, if we give full play to social support, the government will have more time to fight the epidemic.

## 5. Conclusion

During the epidemic, many people are isolated and lack moral support from relatives, friends, and other means. If people cannot effectively obtain enough social support, they will have negative psychological effects. The government and social forces are providing psychological counseling to quarantined people during the COVID-19 epidemic and are obligated to provide psychological crisis intervention and psychological assistance to understand the current psychological status of the quarantined people, accurately meet the needs of psychological services, help the quarantined people to release psychological pressure, and improve their mental health level.

In the context of China’s dynamic clearance, close contacts of COVID-19 have been ordered to be quarantined. How to protect the mental health of the isolated population is the main research issue discussed in this article. Some conclusions can be drawn from this article. Compared with the psychological counseling of social forces, the public under the government’s psychological counseling mode can obtain more psychological benefits. However, with the increase in guidance, the difference between the psychological benefits obtained by the government and the psychological benefits obtained by the guidance of social forces decreases at first and then becomes stable. Under the guidance mode, the social benefits of the government decrease, and the more the guidance, the smaller the social benefits.

The study in this article has some limitations. First, the research background of this article is that China adopts the policy of dynamic zero clearing. However, the dynamic zeroing policy has been suspended since December 2022. Second, this article only considers the situation that there are three ways of psychological counseling. Third, the government has a relatively comprehensive grasp of the infected information in the local area. The research in this article can be extended to some extent. In future studies, the psychological state of the population can be studied when the pandemic is fully unleashed. Meanwhile, it is possible to consider the existence of mixed guidance methods and the failure of the government to fully grasp the infected situation in the region and conduct relevant research.

## Data availability statement

The original contributions presented in the study are included in the article/Supplementary material, further inquiries can be directed to the corresponding author.

## Author contributions

YB is mainly responsible for the work concept or design, drafting the paper, making important modifications to the paper, and approving the final version of the paper to be published, etc. SM is mainly responsible for data collection and data processing. All authors contributed to the article and approved the submitted version.

## Funding

This work was supported by the Doctoral Research Foundation of Shandong University of Management, sdmud2023001.

## Conflict of interest

The authors declare that the research was conducted in the absence of any commercial or financial relationships that could be construed as a potential conflict of interest.

## Publisher’s note

All claims expressed in this article are solely those of the authors and do not necessarily represent those of their affiliated organizations, or those of the publisher, the editors and the reviewers. Any product that may be evaluated in this article, or claim that may be made by its manufacturer, is not guaranteed or endorsed by the publisher.
