# Supplementary material for: From depression to wellbeing: How to protect the mental health of isolated people under the “dynamic clearance” of patients with COVID-19
Source: Front Psychol. 2023 Feb 16;14:1124063. doi: 10.3389/fpsyg.2023.1124063 (PMC9978484; doi:10.3389/fpsyg.2023.1124063)
Supplement: Supplementary file 1 [file Data_Sheet_1.docx]

**Appendix 1**

Derivative of *F_F_*_1_with respect to (13) and derivative of *F_F_*_2_ with respect to (14) respectively, and set it equal to zero, we can get:

$G_{F1}^{\text{*}}\left(t\right)\text{=}\frac{b_{on}}{c_{F1}}$ , $F_{F1}^{\text{*}}\left(t\right)=\frac{b_{off}}{2\lambda}\left(\frac{\partial V_{F1}}{\partial x_{F1}}\right)^{-1}$ (43)

$S_{F2}^{\text{*}}\left(t\right)=\frac{\lambda}{c_{F2}}\left(\frac{\partial V_{F2}}{\partial x_{F2}}\right)$ (44)

By substituting (43) and (44) into the equations (13) and (14), we can get:

$\rho V_{F1}=\left[b_{on}G_{F1}\left(t\right)+\frac{\left(b_{off}\right)^{2}}{2\lambda}\left(\frac{\partial V_{F1}}{\partial x_{F1}}\right)^{-1}-\frac{c_{F1}}{2}\left(\frac{b_{on}}{c_{F1}}\right)^{2}\right.\left.+l_{1}x_{F1}\left(t\right)\right]+\frac{\partial V_{F1}}{\partial x_{F1}}\left[-\lambda\left(\frac{b_{off}}{2\lambda}\right)^{2}\left(\frac{\partial V_{F1}}{\partial x_{F1}}\right)^{-2}+\delta x_{F1}\left(t\right)\right]$ (45)

$\rho V_{F2}=\left[-\frac{c_{F2}}{2}\left(\frac{\lambda}{c_{F2}}\right)^{2}\left(\frac{\partial V_{F2}}{\partial x_{F2}}\right)^{2}\right.\left.+l_{2}x_{F2}\left(t\right)\right]+\frac{\partial V_{F2}}{\partial x_{F2}}\left[\frac{\lambda^{2}}{c_{F2}}\left(\frac{\partial V_{F2}}{\partial x_{F2}}\right)-\delta x_{F2}\left(t\right)\right]$ (46)

Let $V_{F1}^{*}=m_{1}x_{F1}+m_{2}$and $V_{F2}^{\text{*}}=m_{3}x_{F2}+m_{4}$, in which *m*_1_, *m*_2_, *m*_3_ and *m*_4_ are all constants. The parameters of the optimal social welfare function can be obtained by calculation as follows:

$\left\{\begin{matrix} m_{1}=\frac{l_{1}}{\rho-\delta} \\ m_{2}=\frac{1}{\rho}\left[b_{on}G_{F1}\left(t\right)+\frac{\left(b_{off}\right)^{2}}{2\lambda}\left(\frac{l_{1}}{\rho-\delta}\right)^{-1}-\right.\left.\frac{c_{F1}}{2}\left(\frac{b_{on}}{c_{F1}}\right)^{2}\right]+\frac{1}{\rho}\frac{l_{1}}{\rho-\delta}\left[-\lambda\left(\frac{b_{off}}{2\lambda}\right)^{2}\left(\frac{l_{1}}{\rho-\delta}\right)^{-2}\right] \end{matrix}\right.$ (47)

$\left\{\begin{matrix} m_{3}=\frac{l_{2}}{\rho+\delta} \\ m_{4}=-\frac{1}{\rho}\frac{c_{F2}}{2}\left(\frac{\lambda}{c_{F2}}\right)^{2}\left(\frac{l_{2}}{\rho+\delta}\right)^{2}+\frac{1}{\rho}\frac{l_{2}}{\rho+\delta}\frac{\lambda^{2}}{c_{F2}}\left(\frac{l_{2}}{\rho+\delta}\right) \end{matrix}\right.$ (48)

Therefore, it can be concluded that:

$\begin{matrix} V_{F1}^{*}=\frac{l_{1}}{\rho-\delta}x_{F1}+\frac{1}{\rho}\left[b_{on}G_{F1}\left(t\right)+\frac{\left(b_{off}\right)^{2}}{2\lambda}\left(\frac{l_{1}}{\rho-\delta}\right)^{-1}-\right.\left.\frac{c_{F1}}{2}\left(\frac{b_{on}}{c_{F1}}\right)^{2}\right] \\ +\frac{1}{\rho}\frac{l_{1}}{\rho-\delta}\left[-\lambda\left(\frac{b_{off}}{2\lambda}\right)^{2}\left(\frac{l_{1}}{\rho-\delta}\right)^{-2}\right] \end{matrix}$ (49)

$V_{F2}^{\text{*}}=\frac{l_{2}}{\rho+\delta}x_{F2}-\frac{1}{\rho}\frac{c_{F2}}{2}\left(\frac{\lambda}{c_{F2}}\right)^{2}\left(\frac{l_{2}}{\rho+\delta}\right)^{2}+\frac{1}{\rho}\frac{l_{2}}{\rho+\delta}\frac{\lambda^{2}}{c_{F2}}\left(\frac{l_{2}}{\rho+\delta}\right)$ (50)

In this case,

$G_{F1}^{\text{*}}\left(t\right)\text{=}\frac{b_{on}}{c_{F1}}$, $F_{F1}^{\text{*}}\left(t\right)=\frac{b_{off}}{2\lambda}\frac{\rho-\delta}{l_{1}}$ (51)

$S_{F2}^{\text{*}}\left(t\right)=\frac{\lambda}{c_{F2}}\left(\frac{l_{2}}{\rho+\delta}\right)$ (52)

**Appendix 2**

Take the derivative of *F_B_*_1_ and *G_B_*_1_ respectively with respect to (15), and take the derivative of *F_B_*_2_ and *G_B_*_2_ with respect to (16) respectively, and set them equal to zero, we can get:

$G_{G1}^{\text{*}}\left(t\right)\text{=}\frac{b_{on}+\ln\left(1+\beta_{1}\right)}{c_{G1}}$, $F_{G1}^{\text{*}}\left(t\right)\text{=}\frac{-b_{off}}{2\left(-\lambda+k\beta_{1}\right)}\left(\frac{\partial V_{G1}}{\partial x_{G1}}\right)^{-1}$ (53)

$S_{G2}^{\text{*}}\left(t\right)=\frac{1}{c_{G2}}\left[-c_{G2}d\beta_{1}+\frac{\partial V_{G2}}{\partial x_{G2}}\left(\lambda+k\beta_{1}\right)\right]$ (54)

By substituting (53) and (54) into the equations (15) and (16), we can get:

$\begin{matrix} \rho V_{G1}=\left[b_{on}\frac{b_{on}+\ln\left(1+\beta_{1}\right)}{c_{G1}}+\frac{-\left(b_{off}\right)^{2}}{2\left(-\lambda+k\beta_{1}\right)}\left(\frac{\partial V_{G1}}{\partial x_{G1}}\right)^{-1}-\frac{c_{G1}}{2}\left(\frac{b_{on}+\ln\left(1+\beta_{1}\right)}{c_{G1}}\right)^{2}\right. \\ +\frac{b_{on}+\ln\left(1+\beta_{1}\right)}{c_{G1}}\ln\left(1+\beta_{1}\right)\left.+l_{1}x_{G1}\left(t\right)\right] \\ +\frac{\partial V_{G1}}{\partial x_{G1}}\left[\left(-\lambda+k\beta_{1}\right)\left(\frac{-b_{off}}{2\left(-\lambda+k\beta_{1}\right)}\left(\frac{\partial V_{G1}}{\partial x_{G1}}\right)^{-1}\right)^{2}+\delta x_{G1}\left(t\right)\right] \end{matrix}$ (55)

$\begin{matrix} \rho V_{G2}=-\frac{c_{G2}}{2}\left[\frac{1}{c_{G2}}\left(-c_{G2}d\beta_{1}+\frac{\partial V_{G2}}{\partial x_{G2}}\left(\lambda+k\beta_{1}\right)\right)+d\beta_{1}\right]^{2}+l_{2}x_{G2}\left(t\right) \\ +\frac{\partial V_{G2}}{\partial x_{G2}}\left[\left(\lambda+k\beta_{1}\right)\frac{1}{c_{G2}}\left(-c_{G2}d\beta_{1}+\frac{\partial V_{G2}}{\partial x_{G2}}\left(\lambda+k\beta_{1}\right)\right)-\delta x_{G2}\left(t\right)\right] \end{matrix}$ (56)

Let $V_{G1}^{*}=m_{5}x_{G1}+m_{6}$ and $V_{G2}^{\text{*}}=m_{7}x_{G2}+m_{8}$, in which *m*_5_, *m*_6_, *m*_7_, and *m*_8_ are all constants. The parameters of the optimal social welfare function can be obtained by calculation as follows:

$\left\{\begin{matrix} m_{5}=\frac{l_{1}}{\rho-\delta} \\ m_{6}=\frac{1}{\rho}\left[b_{on}\frac{b_{on}+\ln\left(1+\beta_{1}\right)}{c_{G1}}+\frac{-\left(b_{off}\right)^{2}}{2\left(-\lambda+k\beta_{1}\right)}\left(\frac{l_{1}}{\rho-\delta}\right)^{-1}-\frac{c_{G1}}{2}\left(\frac{b_{on}+\ln\left(1+\beta_{1}\right)}{c_{G1}}\right)^{2}\right. \\ +\frac{b_{on}+\ln\left(1+\beta_{1}\right)}{c_{G1}}\left.\ln\left(1+\beta_{1}\right)\right] \\ +\frac{1}{\rho}\frac{l_{1}}{\rho-\delta}\left[\left(-\lambda+k\beta_{1}\right)\left(\frac{-b_{off}}{2\left(-\lambda+k\beta_{1}\right)}\left(\frac{l_{1}}{\rho-\delta}\right)^{-1}\right)^{2}\right] \end{matrix}\right.$ (57)

$\left\{\begin{matrix} m_{7}=\frac{l_{2}}{\rho+\delta} \\ m_{8}=-\frac{c_{G2}}{2}\frac{1}{\rho}\left[\frac{1}{c_{G2}}\left(-c_{G2}d\beta_{1}+\frac{l_{2}}{\rho+\delta}\left(\lambda+k\beta_{1}\right)\right)+d\beta_{1}\right]^{2} \\ +\frac{1}{\rho}\frac{l_{2}}{\rho+\delta}\left[\left(\lambda+k\beta_{1}\right)\frac{1}{c_{G2}}\left(-c_{G2}d\beta_{1}+\frac{l_{2}}{\rho+\delta}\left(\lambda+k\beta_{1}\right)\right)\right] \end{matrix}\right.$ (58)

Therefore, it can be concluded that:

$\begin{matrix} V_{G1}^{*}=\frac{l_{1}}{\rho-\delta}x_{G1}+\frac{1}{\rho}\left[b_{on}\frac{b_{on}+\ln\left(1+\beta_{1}\right)}{c_{G1}}+\frac{-\left(b_{off}\right)^{2}}{2\left(-\lambda+k\beta_{1}\right)}\left(\frac{l_{1}}{\rho-\delta}\right)^{-1}-\frac{c_{G1}}{2}\left(\frac{b_{on}+\ln\left(1+\beta_{1}\right)}{c_{G1}}\right)^{2}\right. \\ +\frac{b_{on}+\ln\left(1+\beta_{1}\right)}{c_{G1}}\left.\ln\left(1+\beta_{1}\right)\right] \\ +\frac{1}{\rho}\frac{l_{1}}{\rho-\delta}\left[\left(-\lambda+k\beta_{1}\right)\left(\frac{-b_{off}}{2\left(-\lambda+k\beta_{1}\right)}\left(\frac{l_{1}}{\rho-\delta}\right)^{-1}\right)^{2}\right] \end{matrix}$ (59) $\begin{matrix} V_{G2}^{\text{*}}=\frac{l_{2}}{\rho+\delta}x_{B2}-\frac{c_{G2}}{2}\frac{1}{\rho}\left[\frac{1}{c_{G2}}\left(-c_{G2}d\beta_{1}+\frac{l_{2}}{\rho+\delta}\left(\lambda+k\beta_{1}\right)\right)+d\beta_{1}\right]^{2} \\ +\frac{1}{\rho}\frac{l_{2}}{\rho+\delta}\left[\left(\lambda+k\beta_{1}\right)\frac{1}{c_{G2}}\left(-c_{G2}d\beta_{1}+\frac{l_{2}}{\rho+\delta}\left(\lambda+k\beta_{1}\right)\right)\right] \end{matrix}$ (60)

In this case,

$G_{G1}^{\text{*}}\left(t\right)\text{=}\frac{b_{on}+\ln\left(1+\beta_{1}\right)}{c_{G1}}$, $F_{G1}^{\text{*}}\left(t\right)\text{=}\frac{-b_{off}}{2\left(-\lambda+k\beta_{1}\right)}\left(\frac{l_{1}}{\rho-\delta}\right)^{-1}$ (61)

$S_{G2}^{\text{*}}\left(t\right)=\frac{1}{c_{G2}}\left[-c_{G2}d\beta_{1}+\frac{l_{2}}{\rho+\delta}\left(\lambda+k\beta_{1}\right)\right]$ (62)

**Appendix 3**

Take the derivatives of *F_M_*_1_ and *G_M_*_1_ respectively with respect to (17), and take the derivatives of *F_M_*_2_ and *G_M_*_2_ respectively with respect to (18), and set them equal to zero, we can get:

$G_{S1}^{\text{*}}\left(t\right)\text{=}\frac{b_{on}+\ln\left(1+\beta_{2}\right)}{c_{G1}}$, $F_{S1}^{\text{*}}\left(t\right)\text{=}\frac{-b_{off}}{2\left(-\lambda+k\beta_{2}\right)}\left(\frac{\partial V_{G1}}{\partial x_{G1}}\right)^{-1}$ (63)

$S_{S2}^{\text{*}}\left(t\right)=\frac{\lambda\text{+}k\beta_{2}}{c_{G2}}\frac{\partial V_{S2}}{\partial x_{S2}}$ (64)

Substituting (67) and (68) into (17) and (18) respectively, we can get:

$\begin{matrix} \rho V_{S1}=\left[b_{on}\frac{b_{on}+\ln\left(1+\beta_{2}\right)}{c_{G1}}+b_{off}\frac{-b_{off}}{2\left(-\lambda+k\beta_{2}\right)}\left(\frac{\partial V_{S1}}{\partial x_{S1}}\right)^{-1}-\frac{c_{S1}}{2}\left(\frac{b_{on}+\ln\left(1+\beta_{2}\right)}{c_{S1}}\right)^{2}\right. \\ \left.+\frac{b_{on}+\ln\left(1+\beta_{2}\right)}{c_{S1}}\ln\left(1+\beta_{2}\right)+l_{1}x_{S1}\left(t\right)\right] \\ +\left.\frac{\partial V_{S1}}{\partial x_{S1}}\left[\left(-\lambda+k\beta_{2}\right)\left(\frac{-b_{off}}{2\left(-\lambda+k\beta_{2}\right)}\right)^{2}\left(\frac{\partial V_{S1}}{\partial x_{S1}}\right)^{-2}+\delta x_{S1}\left(t\right)\right]\right\} \end{matrix}$ (65)

$\rho V_{S2}=\left[-\frac{c_{S2}}{2}\left(\frac{k\beta_{2}}{c_{S2}}\frac{\partial V_{S2}}{\partial x_{S2}}\right)^{2}\right.\left.+l_{2}x_{S2}\left(t\right)\right]+\frac{\partial V_{S2}}{\partial x_{S2}}\left[\left(\lambda+k\beta_{2}\right)\frac{\lambda+k\beta_{2}}{c_{S2}}\frac{\partial V_{S2}}{\partial x_{S2}}-\delta x_{S2}\left(t\right)\right]$ (66)

Let $V_{S1}^{*}=m_{9}x_{S1}+m_{10}$ and $V_{S2}^{\text{*}}=m_{11}x_{S2}+m_{12}$, in which *m*_9_, *m*_10_, *m*_11_ and *m*_12_ are all constants. The parameters of the optimal social welfare function can be obtained by calculation as follows:

$\left\{\begin{matrix} m_{9}=\frac{l_{1}}{\rho-\delta} \\ m_{10}=\frac{1}{\rho}\left[b_{on}\frac{b_{on}+\ln\left(1+\beta_{2}\right)}{c_{S1}}+b_{off}\frac{-b_{off}}{2\left(-\lambda+k\beta_{2}\right)}\left(\frac{l_{1}}{\rho-\delta}\right)^{-1}-\frac{c_{S1}}{2}\left(\frac{b_{on}+\ln\left(1+\beta_{2}\right)}{c_{S1}}\right)^{2}\right. \\ \left.+\frac{b_{on}+\ln\left(1+\beta_{2}\right)}{c_{S1}}\ln\left(1+\beta_{2}\right)\right] \\ +\left[\left(-\lambda+k\beta_{2}\right)\left(\frac{-b_{off}}{2\left(-\lambda+k\beta_{2}\right)}\right)^{2}\left(\frac{l_{1}}{\rho-\delta}\right)^{-2}\right]\frac{l_{1}}{\rho-\delta}\frac{1}{\rho} \end{matrix}\right.$ (67)

$\left\{\begin{matrix} m_{11}=\frac{l_{2}}{\rho+\delta} \\ m_{12}=-\frac{1}{\rho}\frac{c_{S2}}{2}\left(\frac{\lambda+k\beta_{2}}{c_{S2}}\frac{l_{2}}{\rho+\delta}\right)^{2}+\left(\frac{l_{2}}{\rho+\delta}\right)^{2}\frac{1}{\rho}\left(\lambda+k\beta_{2}\right)\frac{\lambda+k\beta_{2}}{c_{S2}} \end{matrix}\right.$ (68)

Therefore, it can be concluded that:

$\begin{matrix} V_{S1}^{*}=\frac{l_{1}}{\rho-\delta}x_{S1}+\frac{1}{\rho}\left[b_{on}\frac{b_{on}+\ln\left(1+\beta_{2}\right)}{c_{S1}}+b_{off}\frac{-b_{off}}{2\left(-\lambda+k\beta_{2}\right)}\left(\frac{l_{1}}{\rho-\delta}\right)^{-1}-\frac{c_{G1}}{2}\left(\frac{b_{on}+\ln\left(1+\beta_{2}\right)}{c_{S1}}\right)^{2}\right. \\ \left.+\frac{b_{on}+\ln\left(1+\beta_{2}\right)}{c_{S1}}\ln\left(1+\beta_{2}\right)\right]+\left[\left(-\lambda+k\beta_{2}\right)\left(\frac{-b_{off}}{2\left(-\lambda+k\beta_{2}\right)}\right)^{2}\left(\frac{l_{1}}{\rho-\delta}\right)^{-2}\right]\frac{l_{1}}{\rho-\delta}\frac{1}{\rho} \end{matrix}$(69)$V_{S2}^{\text{*}}=\frac{l_{2}}{\rho+\delta}x_{S2}-\frac{1}{\rho}\frac{c_{S2}}{2}\left(\frac{\lambda+k\beta_{2}}{c_{S2}}\frac{l_{2}}{\rho+\delta}\right)^{2}+\left(\frac{l_{2}}{\rho+\delta}\right)^{2}\frac{1}{\rho}\left(\lambda+k\beta_{2}\right)\frac{\lambda+k\beta_{2}}{c_{S2}}$ (70)

In this case,

$G_{S1}^{\text{*}}\left(t\right)\text{=}\frac{b_{on}+\ln\left(1+\beta_{2}\right)}{c_{S1}}$, $F_{S1}^{\text{*}}\left(t\right)\text{=}\frac{-b_{off}}{2\left(-\lambda+k\beta_{2}\right)}\left(\frac{l_{1}}{\rho-\delta}\right)^{-1}$ (71)

$S_{S2}^{\text{*}}\left(t\right)=\frac{\lambda+k\beta_{2}}{c_{S2}}\frac{l_{2}}{\rho+\delta}$ (72)
